# Association between obesity and psychological distress in the Chilean population during the COVID-19 pandemic: Social Wellbeing Survey 2021

**DOI:** 10.1371/journal.pone.0333697

**Published:** 2025-11-03

**Authors:** Carlos González-Torres, Lydia Lera, Pablo A. Lizana

**Affiliations:** 1 Pontificia Universidad Católica de Valparaíso, Valparaíso, Chile; 2 Latin Division, Keiser University, Fort Laurderdale, Florida, United States of America; 3 Laboratory of Epidemiology and Morphological Sciences, Pontificia Universidad Católica de Valparaíso, Valparaíso, Chile; Prague University of Economics and Business: Vysoka Skola Ekonomicka v Praze, CZECHIA

## Abstract

The COVID-19 pandemic has exacerbated obesity and mental health problems, particularly anxiety and depression. Both conditions share common risk factors, suggesting a possible bidirectional relationship. This study analyses the association between obesity and psychological distress in the Chilean population during the pandemic. A secondary analysis of data from the 2021 Social Wellbeing Survey (n = 10395) was conducted using logistic regression models to examine the relationship between obesity and the presence of severe psychological distress. The prevalence of obesity and severe psychological distress was higher in women (31.85% and 7.66%) than in men (25.1% and 3.6%). Individuals with obesity had a higher risk of severe psychological distress OR 1.3 (95% CI 1.05–1.60), as did women OR 2.16 (95% CI 1.83–2.65). Conversely, individuals with severe psychological distress had a higher risk of obesity OR 1.4 (95% CI 1.19–1.71), as did women OR 1.4 (95% CI 1.26–1.51) and individuals couple/married OR 1.3 (95% CI 1.17–1.46). Additionally, higher educational levels are a protective factor for both obesity and severe psychological distress. A higher prevalence of obesity and psychological distress was observed in women and variations by age. Obesity and severe psychological distress behaved as mutual risk factors, suggesting a possible bidirectional relationship. These findings support the need for mental health interventions for at-risk groups.

## Introduction

In 2022, 33.7% of women and 27.6% of adult men in Chile lived with obesity [1]. This information is a reality which had been present in the country since well before the COVID-19 pandemic. Reports from the National Health Surveys, including data from 2009 to 2016, already showed obesity in Chile rising from 25% to 34% of the adult population during that timeframe [2], along with children [3]. This progressive rise in obesity could be due to unhealthy lifestyle changes [4–6] which increased during pandemic lockdowns [7], since one core healthy habit to control body weight that dropped dramatically was physical activity [8,9]. This increase was mainly due to several factors that emerged during quarantine. First, the closure of gyms and limited access to parks significantly reduced opportunities for physical activity. Second, many individuals experience changes in their eating patterns. Lastly, teleworking and the increased use of technological devices contributed to a more sedentary lifestyle [10–12].

Along with widespread lifestyle changes during the pandemic, there was a sharp rise in uncertainty related to health, social, and economic conditions, which contributed to a global increase in mental health problems [13–16]. Individuals with pre-existing health conditions, such as obesity, may have been particularly vulnerable to these effects. Although the mechanisms are not yet fully understood, growing evidence supports a bidirectional relationship between obesity and mental health problems, likely mediated by shared risk factors such as poor diet, physical inactivity, genetic predispositions, pharmacological treatments, and imbalances in specific neurotransmitters [17–21]. These common pathways have been linked to various disorders, including depression, anxiety, eating disorders, and post-traumatic stress. Within this relation between obesity and mental problems, another considered factor may be psychological distress, which can be defined as a state of emotional suffering which can be caused by diverse factors including trauma, anxiety, depression, or stress, and which can affect quality of life (QoL) for people if it continues over a long period [22–24]. Although it is not specifically mentioned within the bi-directional relation, psychological distress can be an important variable to consider in the relation between obesity and mental problems.

In this sense, there is a lack of literature approaching the relation between psychological distress and obesity, given that distress is not considered a mental illness on account of being a response to a stressor, leading it to be considered a symptom which most people experience at some point in their lives [25]. The problem arises when distress takes place in a prolonged way, increasing the probability of suffering from cardiovascular, infectious, and mental illnesses. While stress is a normal response in our bodies, this can reach a point which ultimately affects the health of its sufferers [26–29]. Within this context, greater understanding of the relation between obesity and distress could help develop preventive health interventions to improve QoL for obese people and halt the rise of new pathologies.

During the COVID-19 pandemic, Chile had two studies about psychological distress levels based upon the PHQ-4 instrument. The first of these studies was conducted during the initial wave of COVID-19 (30 May to 10 June 2020), and its objective was to estimate how social, economic, and domestic effects are related to psychological distress within the population. It indicated that being a woman, persistently feeling lonely, living in the areas of the country most affected by the pandemic, and the loss of income due to work stoppages arising from quarantine were significantly associated with high psychological distress levels [30]. The second study is a longitudinal study based upon the aforementioned results, whose objective was to compare distress levels during the first and second wave of COVID-19 in 2020 (30 May-10 June and 15 September-9 October), where distress levels rose from 22.6% to 27%, particularly among women, people who reported feeling lonely, urban zone residents, and overcrowded households, amongst others [31]. The fact that both studies covered the first waves of COVID-19 in Chile indicates how, as infections rose, distress levels also rose and had a greater effect on specific population groups including women and urban residents.

In South America, Brazil compared psychological distress levels before and during the pandemic but also incorporated its relationship with eating and physical health patterns. Lockdowns and the lifestyle changes which they drove affected the mental health of the population compared with those who remained active and ate healthy food [10], reaffirming the idea that regular physical activity and proper diet could be key protective factors against developing psychological distress, even in contexts of high uncertainty such as the COVID-19 pandemic [8,9,14,15].

However, previous studies on the relationship between obesity and psychological distress have reported inconsistent findings. Some have found no significant associations [32], others have identified a positive relationship [10,33], and several suggest that this association may depend on additional factors such as diet or pre-existing psychiatric conditions [34]. Therefore, the present study aims to analyze the bidirectional relationship between obesity and psychological distress in the Chilean population during the COVID-19 pandemic. We hypothesize that individuals with obesity have a higher risk of experiencing psychological distress and, conversely, that psychological distress may increase the risk of obesity, particularly given the pandemic’s impact on mental health.

## Methods

A secondary analysis was carried out based upon the database from the Social Wellbeing Survey (SWS) 2021 carried out by the Chilean Social Development and Family Ministry. SWS has a sample size of 10921 Chileans over age 18, and its main objective is to have a battery of social wellbeing indicators in 11 dimensions: Subjective wellbeing, Education, Work, Income, Work-life balance, social relations, Civic commitment and governance, Health, Housing, Environmental quality, and Physical safety [35]. Given its multidimensional scope, this dataset offers a solid foundation for analyzing the association between obesity and psychological distress within a broad sociodemographic and health-related framework, as proposed in the present study. The sampling method of the SWS was probabilistic, two-phase, and stratified. The first phase corresponds to dwellings and households whose sample design is probabilistic, stratified, by clusters, and in multiple stages; then, the second phase is obtained from persons 18 years of age and older through random and stratified sampling by the 16 regions of Chile. The unit of selection is the person. The representativeness of the SWS is national, by geographic zones (urban-rural), and regional (16 regions of Chile). The evaluation period for participants was between April and May 2021.The response rate of the SWS was 53.9%, and the refusal rate was 13.4% [35]. The data were obtained from public sources of the Government of Chile, Ministry of Social Development of Chile [35].

### Participants

**Fig 1:**
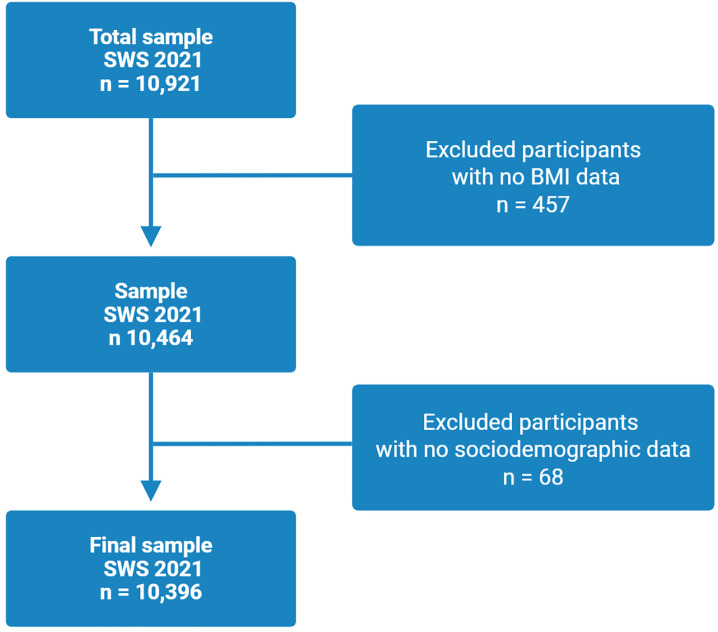
Flowchart of participants with indications of the number of excluded by variables. (SWS: Social Wellbeing Survey; BMI: Body Mass Index).

From the total sample of 10,921 individuals included in the SWS 2021, we selected participants based on the availability of complete data for the variables analyzed in this study. The final sample was defined after excluding respondents with missing values for key sociodemographic and health-related variables. Fig 1 illustrates the flow of exclusions and the resulting analytic sample.

The variables considered in this analysis included gender, age, marital status, educational level, and area of residence. For the latter, urban areas were defined as those with a population density of at least 50,000 inhabitants, whereas rural areas were defined as having fewer than 5,000 inhabitants. Age was operationalized as a continuous and categorical variable using the predefined age groups from the SWS (18–25, 30–44, 45–59, and 60 years old or more). Age was included as a categorical variable for the logistic regression models. Educational levels were grouped into three categories (up to 8 years, up to 12 years, and >13 years) along with marital status (single, married/partnered, divorced/widowed).

### Variable selection

The first variable selected was the psychological distress of the Chilean population within the “Health” dimension of the SWS. Psychological distress was measured and categorized using the Patient Health Questionnaire-4 (PHQ4), consisting of 4 questions to evaluate the moods reported by respondents in order to detect the gravity of anxiety and depression symptoms along with overall perceptions of psychological distress. Despite not having been validated for the Chilean population, it has already been used previously for studies within this group as well as within the COVID-19 pandemic context [30,31].

The second variable chose was BMI, calculated by the weight and height reported by the Chilean population which participated in the SWS 2021. The calculation was done using the formula of weight in kilograms/ height in meters squared and classifying the result according to the following criteria from the World Health Organization: Under-weight <18.5 kg/m2, Normal weight 18.50–24.99 kg/m2, Overweight ≥ 25 kg/m2, Obese ≥ 30 kg/m2 [36,37].

### Statistical analyses

Data were analyzed using STATA 16 for Windows. Descriptive statistics were used to characterize the sample. Categorical variables were summarized using frequencies and percentages (n, %), while continuous variables were described using means and standard deviations (M ± SD).

The Chi-squared test was applied for bivariate analyses to assess associations between categorical variables. Comparisons between continuous variables were performed using non-parametric tests due to the non-normal distribution of the data as indicated by the Kolmogorov-Smirnov test. Specifically, the Mann-Whitney U test was used for comparisons between two groups, and the Kruskal-Wallis test, followed by Dunn’s post-hoc test, was used for comparisons involving three or more groups. To assess the magnitude of observed differences, effect sizes were calculated using Cramer’s V for categorical variables, Cohen’s d and Eta-Squared for continuous variables. The effect size was interpreted based on widely accepted thresholds: values between 0.10 and 0.29 were considered weak, between 0.30 and 0.49 moderate, and values equal to or greater than 0.50 were classified as strong.

To examine the bidirectional relationship between obesity and psychological distress, we conducted six logistic regression models. The dependent variable in the first three models was severe psychological distress, and the independent variable was the BMI category. In the remaining three models, the dependent variable was obesity, and the independent variable was the level of psychological distress. This rotation of the dependent and independent variables allowed us to explore the bidirectional association between both conditions.

All regression models were adjusted for key sociodemographic variables: gender, age, area of residence, and educational level. Model fit was evaluated using the Hosmer-Lemeshow test, with p-values greater than 0.05 indicating good model fit. Results from the logistic regression models are presented as Odds Ratios (OR) with 95% Confidence Intervals (CI). Statistical significance was set at an alpha level of 0.05 for all analyses.

## Results

Table 1 presents the associations between BMI, psychological distress, and sociodemographic characteristics in a Chilean population sample stratified by gender. The total sample consisted of 10,396 participants, of whom 4,446 were men (42.77%) and 5,950 were women (57.23%). Statistically significant associations were observed between gender and BMI, psychological distress, age, and marital status (p < 0.001). Regarding BMI, a higher prevalence of obesity was found among women compared to men (p < 0.001). Similarly, a greater proportion of women reported experiencing mild, moderate, and severe psychological distress compared to men (p < 0.001).

**Table 1:** Comparison of sociodemographic characteristics, body mass index, and psychological distress by gender in the study population (N = 10,396).

Variables	Total sample *(N = 10,396)*	Male (*N = 4,446*)	Female (*N = 5,950*)	p-value	Effect size
	n, (%)	n, (%)	n, (%)		
Age (years)		45.99 ± 18.01	46.74 ± 17.20	0.031	0.043^e^
18-29	2231 (22.42)	1084 (24.38)	1247 (20.96)	<0.001^b^	0.047^f^
30-44	2512 (24.16)	1035 (23.28)	1477 (24.82)		
45-59	2829 (27.21)	1141 (25.66)	1688 (28.37)		
> 60	2724 (26.20)	1186 (26.68)	1538 (25.85)		
Marital Status				<0.001^b^	0.145^f^
Single	4186 (40.27)	1788 (40.22)	2398 (40.30)		
Couple/Married	4609 (44.33)	2224 (50.02)	2385 (40.08)		
DWW^c^	1601 (15.40)	434 (9.76)	1167 (19.61)		
Educational level				0.478^b^	0.012^f^
Low	3511 (33.77)	1473 (33.13)	2038 (34.25)		
Medium	4557 (43.83)	1972 (44.35)	2585 (43.45)		
High	2328 (22.39)	1001 (22.51)	1327 (22.30)		
Residence Area				0.038^b^	0.020^f^
Urban	8874 (85.36)	3758 (84.53)	5116 (85.98)		
Rural	1522 (14.64)	688 (15.47)	834 (14.02)		
Body mass index				<0.001^b^	0.098^f^
Under weight	115 (1.11)	41 (0.92)	74 (1.24)		
Normal weight	2969 (28.56)	1213 (27.28)	1756 (29.51)		
Overweight	4301 (41.37)	2076 (46.69)	2225 (37.39)		
Obese	3011 (28.96)	1116 (25.10)	1895 (31.85)		
Psychological distress^d^				<0.001^b^	0.177^f^
No symptoms	4683 (45.05)	2434 (54.75)	2249 (37.80)		
Mild symptoms	3827 (36.81)	1431 (32.29)	2396 (40.27)		
Moderate symptoms	1270 (12.22)	421 (9.47)	849 (14.27)		
Severe symptoms	616 (5.93)	160 (3.60)	456 (7.66)		

^a^Mann-Whitney test.

^b^Chi squared.

^c^Divorced-Widowed.

^d^Based on PHQ4 questionnaire.

^e^Data are expressed as mean and standard deviation.

^d^Cohen’s d.

^f^Cramer’s V.

Table 2 presents the associations between sociodemographic characteristics and psychological distress across BMI categories. Age, considered a continuous variable, showed a positive association with BMI; individuals with obesity had a higher mean age than those with normal weight or overweight (p < 0.001). BMI was also significantly associated with marital status, educational level, area of residence, and psychological distress (p < 0.001). Specifically, severe psychological distress was more prevalent among individuals with obesity, while moderate and mild symptoms were more commonly reported by those with overweight (p < 0.001).

**Table 2:** Associations between BMI with sociodemographic characteristics and psychological distress of the population.

Variables	Body Mass Index				p-value	Effect size
	Under weight^1^	Normal weight^2^	Overweight^3^	Obese^4^		
	n, (%)	n, (%)	n, (%)	n, (%)		
Age (years)^e^	39.84 ± 21.33	43.44 ± 19.18	47.68 ± 17.06	47.81 ± 15.93	<0.001ª (4> 1,2,3; 3 > 1,2; 2 > 1)	0.003^e^
18-29	58 (50.43)	991 (33.38)	800 (18.60)	482 (16.01)	<0.001^b^	0.113^g^
30-44	19 (16.52)	626 (21.08)	1085 (25.23)	782 (25.97)		
45-59	10 (8.70)	629 (21.19)	1213 (28.20)	977 (32.45)		
> 60	28 (24.35)	723 (24.35)	1203 (27.97)	770 (25.57)		
Marital Status					<0.001^b^	0.145^g^
Single	75 (65.22)	1537 (51.77)	1545 (35.92)	1029 (34.17)		
Couple/Married	24 (20.87)	1011 (34.05)	2055 (47.78)	1519 (50.45)		
DWW^c^	16 (13.91)	421 (14.18)	701 (16.30)	463 (15.38)		
Educational level					<0.001^b^	0.093^g^
Low	28 (24.35)	781 (26.31)	1460 (33.95)	1242 (41.25)		
Medium	66 (57.39)	1455 (49.01)	1795 (41.73)	1241 (41.22)		
High	21 (18.26)	733 (24.69)	1046 (24.32)	528 (17.54)		
Residence Area					<0.001^b^	0.051^g^
Urban	101 (87.83)	2604 (87.71)	3669 (85.31)	2500 (83.03)		
Rural	14 (12.17)	365 (12.29)	632 (14.69)	511 (16.97)		
Psychological distress^d^					<0.001^b^	0.039^g^
No symptoms	38 (33.04)	1323 (44.56)	2046 (47.57)	1276 (42.38)		
Mild symptoms	48 (41.74)	1125 (37.89)	1540 (35.81)	1114 (37.00)		
Moderate symptoms	24 (20.87)	345 (11.62)	501 (11.65)	400 (13.28)		
Severe symptoms	5 (4.35)	176 (5.93)	214 (4.98)	221 (7.34)		

^a^Kruskal-Wallis test with Dunn’s post hoc; superscripts 1, 2, 3 and 4 represent the compared body mass index groups.

^b^Chi squared.

^c^Divorced-Widowed.

^d^Based on PHQ4 questionnaire.

^e^Data are expressed as mean and standard deviation.

^f^Eta-squared.

^g^Cramer’s V.

Table 3 displays the associations between psychological distress categories and sociodemographic variables. Significant associations were found between psychological distress and age, marital status, educational level, and area of residence (p < 0.001). Psychological distress symptoms were more severe among younger participants, with symptom severity decreasing progressively with increasing age (p < 0.001). However, the observed effect sizes are weak (Tables 1–3).

**Table 3:** Associations between psychological distress and sociodemographic characteristics of the population.

Variables	Psychological distress				p-value	Effect-Size
	No symptoms^1^	Mild^2^	Moderate^3^	Severe^4^		
	n, (%)	n, (%)	n, (%)	n, (%)		
Age (years)^d^	47.46 ± 17.56	45.70 ± 17.58	45.45 ± 17.27	44.97 ± 17.50	0.001a (1 > 2,3,4; 2 > 3,4; 3 > 4)	0.014^e^
18-29	937 (20.01)	913 (23.86)	308 (24.25)	173 (28.08)	<0.001^b^	0.041^f^
30-44	1165 (24.88)	929 (24.27)	291 (22.91)	127 (20.62)		
45-59	1251 (26.71)	1027 (26.84)	382 (30.08)	169 (27.44)		
> 60	1330 (28.40)	958 (25.03)	289 (22.76)	147 (23.86)		
Marital Status					<0.001^b^	0.058^f^
Single	1776 (37.92)	1588 (41.49)	545 (42.91)	277 (44.97)		
Couple/Married	2267 (48.41)	1621 (42.36)	491 (38.66)	230 (37.34)		
DWW^c^	640 (13.67)	618 (16.15)	234 (18.43)	109 (17.69)		
Educational level					0.001^b^	0.033^f^
Low	1545 (32.99)	1283 (33.52)	462 (36.38)	221 (35.88)		
Medium	2011 (42.94)	1698 (44.37)	570 (44.88)	278 (45.13)		
High	1127 (24.07)	846 (22.11)	238 (18.74)	117 (18.99)		
Residence Area					0.002^b^	0.037^f^
Urban	3930 (83.92)	3308 (86.44)	1105 (87.01)	531 (86.20)		
Rural	753 (16.08)	519 (13.56)	165 (12.99)	85 (13.80)		

^a^Kruskal-Wallis test with Dunn’s post hoc; superscripts 1, 2, 3 and 4 represent the distress groups compared.

^b^Chi squared.

^c^Divorced-Widowed.

^d^Data are expressed as mean and standard deviation.

^e^Eta-squared.

^f^Cramer’s V.

Table 4 summarizes the logistic regression results assessing the association between psychological distress, BMI, and sociodemographic characteristics. Within the BMI categories, only obesity was significantly associated with an increased risk of psychological distress in Model 3, which was adjusted for sociodemographic variables (OR: 1.3; 95% CI: 1.05–1.60). Additionally, being female (OR: 2.2; 95% CI: 1.83–2.65) and being aged 18–25 years (OR: 1.5; 95% CI: 1.21–1.87) were associated with higher odds of experiencing psychological distress. In contrast, the age groups 30–44, 45–59, and 60 + showed a protective effect against severe psychological distress compared to the 18–25 reference group, as did having a high level of education (OR: 0.76; 95% CI: 0.59–0.98).

**Table 4:** Logistic regression for the relationship between the presence of severe psychological distress according to body mass index and sociodemographic characteristics.

	Severe psychological distress		
	Model 1	Model 2	Model 3
	OR (95%CI)	OR (95%CI)	OR (95%CI)
**Body mass index**			
Normal weight	REF	REF	REF
Under weight	0.69 (0.28 - 1.73)	0.65 (0.26 - 1.62)	0.64 (0.26 - 1.60)
Overweight	0.88 (0.71 - 1.08)	0.93 (0.75 - 1.14)	0.93 (0.75 - 1.14)
Obese	1.23 (0.99 - 1.51)	1.31 (1.06 - 1.61)*	1.30 (1.05–1.60)*
**Gender**			
Male	REF	REF	REF
Female	2.17 (1.80–2.61)***	2.21 (1.83 - 2.65)***	2.16 (1.83–2.65)***
**Age (years)**			
18-29		REF	REF
30-44		0.62 (0.49 - 0.79)***	0.65 (0.50 - 0.84)***
45-59		0.72 (0.58 - 0.90)***	0.72 (0.55 - 0.94)*
> 60		0.68 (0.54 - 0.85)***	0.64 (0.47 - 0.86)***
**Marital Status**			
Single			REF
Couple/Married			0.86 (0.69 - 1.06)
DWW^c^			1.05 (0.80 - 1.38)
**Educational level**			
Low			REF
Medium			0.84 (0.68 - 1.04)
High			0.76 (0.59 - 0.98)*
**Residence Area**			
Rural			REF
Urban			1.08 (0.85 - 1.37)
**Hosmer-Lemeshow** ^a^	0.659	0.675	0.824

^a^Values above 0.05 indicate that the model fits the data.

*p < .05, **p < .01, ***p < .001.

Table 5 presents the logistic regression results examining the association between obesity and psychological distress and sociodemographic characteristics. The analysis shows that moderate and severe psychological distress are significantly associated with increased odds of obesity (OR: 1.2; 95% CI: 1.01–1.33 and OR: 1.4; 95% CI: 1.19–1.71, respectively). Additionally, being female was associated with a higher likelihood of obesity (OR: 1.4 95% CI: 1.26–1.51). Higher levels of education appeared to be protective: individuals with medium and high educational attainment had significantly lower risk of obesity (OR: 0.73; 95% CI: 0.65–0.81 and OR: 0.52; 95% CI: 0.45–0.59, respectively).

**Table 5:** Logistic regression for the relationship between obesity according to psychological distress and sociodemographic characteristics.

	Obesity		
	Model 1	Model 2	Model 3
	OR (95%CI)	OR (95%CI)	OR (95%CI)
**Psychological distress**			
No symptoms	REF	REF	REF
Mild symptoms	1.05 (0.95–1.15)	1.07 (0.97 - 1.18)	1.06 (0.96 - 1.17)
Moderate symptoms	1.16 (1.01 - 1.33)*	1.18 (1.03 - 1.35)*	1.16 (1.01 - 1.33)*
Severe symptoms	1.38 (1.16 - 1.66)***	1.45 (1.21 - 1.73)***	1.43 (1.19 - 1.71)***
**Gender**			
Male	REF	REF	REF
Female	1.36 (1.25 - 1.49)***	1.33(1.22 - 1.98)***	1.38 (1.26 - 1.51)***
**Age (years)**			
18-29		REF	REF
30-44		1.74(1.52 - 1.98)***	1.61 (1.40 - 1.86)***
45-59		2.01 (1.77 - 2.28)***	1.61 (1.38 - 1.87)***
> 60		1.52 (1.34 - 1.74)***	1.09 (0.93 - 1.29)
**Marital Status**			
Single			REF
Couple/Married			1.31 (1.17 - 1.46)***
DWW^c^			0.99 (0.86 - 1.51)
**Educational level**			
Low			REF
Medium			0.73 (0.65 - 0.81)***
High			0.52 (0.45 - 0.59)***
**Residence Area**			
Rural			REF
Urban			0.89 (0.79 - 0.99)*
**Hosmer-Lemeshow** ^a^	0.394	0.176	0.338

^a^Values above 0.05 indicate that the model fits the data

*p < .05, **p < .01, ***p < .001

Regarding age, the categories 30–44 and 45–59 years were significantly associated with a higher risk of obesity (OR: 1.61; 95% CI: 1.40–1.86 and OR: 1.61; 95% CI: 1.38–1.87, respectively), in comparison to the 18–29 reference group. Similarly, being married was associated with greater odds of obesity (OR: 1.31; 95% CI: 1.17–1.46), whereas residing in urban areas remained a protective factor (OR: 0.89; 95% CI: 0.79–0.99).

## Discussion

The objective of the present study was to analyze the bi-directional relation between obesity and psychological distress within the Chilean population during the COVID-19 pandemic. A significantly greater prevalence of obesity and psychological distress was reported among women compared with men. Concerning BMI and its association with distress, we observed that obese people had a higher rate of severe psychological distress, while overweight people had a greater prevalence of moderate psychological distress. Logistic regression analyses also reported that obesity is a risk factor for severe psychological distress, while moderate and severe psychological distress are risk factors for obesity, two findings which provide evidence of a possible bi-directional relation between both variables.

The differences in observed obesity rates were greater in women than in men (see Table 1), which is a widely reported observation within the literature, where female obesity is attributed to hormonal, socioeconomic, and even cultural factors [38–40]. Females have also been described as more physically inactive compared with men with regards to moderate-vigorous activities [41,42], so that within a pandemic context where physical activity levels fell drastically [8,9], we can expect weight gain among women during the pandemic.

Concerning the associations between BMI and sociodemographic characteristics of the Chilean population (see Table 2), one notable finding is the positive association between age and BMI, with individuals classified as obese presenting the highest average age. This pattern may reflect a combination of morpho-physiological changes associated with aging, including a progressive decline in muscle mass (sarcopenia), basal metabolic rate, and overall energy expenditure, which in turn can reduce mobility and physical capacity [43,44]. Although sarcopenia alone may contribute to a lower lean body mass, it is often accompanied by increased fat mass and redistribution of adipose tissue, particularly visceral fat, leading to higher BMI values despite muscle loss. These dynamics help explain why older adults may be more likely to fall into higher BMI categories, even when total body weight changes are not pronounced [43]. About educational level, several studies have reported that higher educational attainment is associated with a lower risk of being overweight and obesity [45]. This protective effect may be attributed to education’s role in promoting healthier behaviors and reducing exposure to risk factors such as physical inactivity, sedentary lifestyles, and the consumption of alcohol and tobacco [46–48]. Consistent with this evidence, our findings suggest that having a higher educational level is a protective factor when compared to having a low educational level (see Table 5). Finally, the higher obesity rate in urban zones compared with rural areas may be due to these zones always having reported low physical activity levels, higher consumption of food high in fats and refined sugars, more sedentary lifestyles due to transportation methods, and the recent rise of teleworking which took shape during the pandemic [11,12,49,50].

The associations observed between BMI and sociodemographic characteristics align with other results from the last years before the pandemic within the Chilean population, where the population groups with the highest obesity rates were older, female, and less educated [2,51]. Even so, we can note how the pandemic, mainly because of lifestyle changes which it generated, was able to increase these obesity patterns’ prevalence within the country.

Psychological distress was also significantly associated with sociodemographic characteristics. Women reported higher levels of mild, moderate, and severe distress compared to men (see Table 1). Previous studies conducted during the pandemic have similarly shown that women experienced elevated levels of stress, depression, and anxiety [16,28,52]. This could be explained by the specific social factors of the gender, which for a long time have determined higher rates of mental disorders among women than men [53,54]. For educational level (see Table 3), the results from our study show that low and medium educational levels present a higher rate of moderate and severe symptoms of psychological distress. These results are similar to those reported in other studies within a pandemic context, which also identified people with lower education levels as having higher stress rates [55,56]. This relation between a low education level and the presence of psychological malaise could be explained by these people having fewer job opportunities, increasing economic uncertainty [57], which also limits access to mental health services along with seeking information about symptoms and treatments [58,59]. When examining marital status in the context of a pandemic, it is reasonable to expect that single individuals would experience higher levels of distress. This is because having a partner provides significant emotional support, and the absence of such support during lockdowns may have greatly impacted distress levels among this group [60].

In the regression models (see Tables 4 and 5) we can observe a bidirectional relation between severe psychological distress and obesity, with each acting as a risk factor for the other. The classification of distress as a symptom rather than a disorder may be the reason for its exclusion from the articles on relations between BMI and mental disorders [61,19]. Even so, it is important to have greater emphasis on distress, given that if it continues for prolonged timeframes it can generate mental disorders in people and affect their QoL [22–24]. The reasons for the existence of this bi-directional relation between stress and obesity may follow the same logic as the bi-directional relation between mental disorders and obesity, where there are common underlying factors (physical activity, diet, genetics, and consuming medications) which, by promoting the development of one affliction, can also encourage the other [10,33,62].

Obesity is associated with high morbidity and mortality [63,64], as well as various psychological disorders [21]. In turn, psychological distress can also encourage the development of various psychological disorders, which along with their treatments lead to overeating and weight gain [65–67]. Therefore, obesity and distress are separately capable of generating psychological disorders, and biological aspects like physical activity, diet, and particularly hormonal imbalances can generate both obesity and psychological distress [65,68,69]. Psychological distress can thus be a crucial factor for developing obesity and vice-versa, and both can be important factors for developing psychological disorders.

These findings take on greater importance in the context of the COVID-19 pandemic, where both mental and physical health in the population were affected by uncertainty over public health and the lifestyle changes generated by lockdown [70,71]. Many pandemic-era lifestyle changes decreased QoL for people [72,73], and are also highly associated with increasing obesity and psychological malaise levels. Among these, we can highlight the decreased physical activity patterns, social isolation, consuming more highly processed foods, and more sedentary behavior [74–77].

Therefore, effective mental health interventions require a deeper understanding of the relationship between obesity and psychological distress, which functions both as a symptom and a potential risk factor for the development of mental disorders and other health conditions. This would help create the opportunity to improve mental health in obese people and reduce the risk of them developing mental disorders in the future, by ensuring that all preventive approaches carried out on the basis of this relation can have a significant impact on QoL and general wellbeing amongst affected people. The preceding point is even more relevant considering how the literature shows that existing mental health interventions have a good quality-price ratio with different approaches [78,79]. The problem is that these are mainly present within high-income countries, meaning that in the case of Chile, a country with historic socioeconomic inequalities [80,81], it is necessary to analyze extant national mental health policies and how including distress within them can improve QoL, particularly for obese people, given that they appear to be more vulnerable to various mental disorders as well as their associated symptoms.

### Limitations and strengths

The first limitation is due to the cross-sectional nature of the present study, which lets us obtain data from people in a given moment while not letting us follow changes in this data. Furthermore, only having BMI as a body variable is a major study limitation, as it does not consider other relevant factors including body fat percentages, body fat distribution, or muscle mass in each subject [82,83]. In addition, small effect sizes reported may be due to the lack of weighting and stratification of the probability sample. Nevertheless, the strengths include strong national representation of the results due to database sample size, psychological distress classification by intensity, and the pandemic context which gave rise to this dataset.

## Conclusion

Through the secondary analysis of the Social Wellbeing Survey 2021 during the COVID-19 health crisis, we can report that both obesity and psychological distress are more prevalent in women and people with low educational levels. Young adults also had the highest psychological distress level; by contrast, older people had the highest obesity levels. We also observed that both psychological distress and obesity act as risk factors for each other, which could lead us to assume the existence of a bi-directional relation between both conditions in the Chilean population during the COVID-19 pandemic. We thus propose that it is necessary to continue researching this relation, since better comprehension of the obesity-psychological distress relation could help develop new health interventions in order to improve public policies as well as mental health for obese people, thereby reducing their future risk of developing mental disorders.
